# Survey of bacteria associated with septic arthritis in beef feedlot cattle

**DOI:** 10.1128/aem.01675-25

**Published:** 2026-01-21

**Authors:** Daniel Kos, Brian Warr, Danae M. Suchan, Danilo Wadt, Jennifer N. Russell, Mallory Norfield, Jenny Liang, Murray Jelinski, Andrew D. S. Cameron, Antonio Ruzzini

**Affiliations:** 1 Department of Biology, University of Regina 98642, Regina, Saskatchewan, Canada; 2 Faculty of Science, University of Regina, Institute for Microbial Systems and Society 98641, Regina, Saskatchewan, Canada; 3 Department of Large Animal Clinical Sciences, Western College of Veterinary Medicine, University of Saskatchewan 70399, Saskatoon, Saskatchewan, Canada; 4 Veterinary Agri-Health Services Ltd, Airdrie, Alberta, Canada; 5 Department of Veterinary Microbiology, Western College of Veterinary Medicine, University of Saskatchewan 7235, Saskatoon, Saskatchewan, Canada; 6 Department of Biochemistry, Microbiology and Immunology, College of Medicine, University of Saskatchewan 12371, Saskatoon, Saskatchewan, Canada; Universidad de los Andes, Bogotá, Colombia

**Keywords:** antimicrobial resistance (AMR), feedlot, cattle, *Metamycoplasma alkalescens*, *Mycoplasmopsis bovis*, targeted metagenomics, septic arthritis

## Abstract

Septic arthritis (SA) is a cause of lameness in cattle attributed to bacterial infections. *Mycoplasmopsis bovis* is the best known and characterized etiological agent of SA; however, cases caused by diverse bacteria have been reported. Accordingly, we surveyed bacteria associated with septic and healthy joints from animals in western Canadian feedlots. Microbial community profiling showed that *M. bovis* was the most frequently detected pathogen in septic joints, followed by *Metamycoplasma alkalescens* and *Trueperella pyogenes*. In most cases, disease was ostensibly caused by a single pathogen, though polymicrobial infections and complex communities were also observed in DNA isolated from septic joints. The application of enhanced metagenomics by target DNA hybridization capture sequencing (CapSeq) provided more robust pathogen detection and characterization. CapSeq revealed resistance determinants that escaped detection using a conventional shotgun metagenomic approach. Notably, a series of nucleotide polymorphisms to *M. bovis rrs*, *rrl*, *gyrA*, and *parC* gene sequences were observed that confer resistance to macrolides and oxytetracycline-resistant *T. pyogenes* were also apparent in the CapSeq data. Complementary pathogen isolation, whole-genome sequencing, and phenotyping efforts were focused on the two most prominent pathogens, *M. bovis* and *M. alkalescens*, and corroborated metagenomic data sets.

**IMPORTANCE:**

Informed antimicrobial use for the treatment of septic arthritis (SA) has been limited by overlooking the potential diversity of causative agents and our knowledge of their antimicrobial resistance (AMR) profiles. This survey begins to provide epidemiological insights, offering renewed appreciation of *Metamycoplasma alkalescens* as an etiological agent of SA and highlighting the prominence of important AMR determinants. Finally, the survey suggests that our knowledge of even the identities of the causative agents of SA is incomplete.

## INTRODUCTION

Septic arthritis (SA) is broadly defined as an infectious disease of joints associated with inflammation. SA falls under the clinical umbrella of animal lameness and, in the beef production system, it occurs most frequently in calves that enter feedlots in fall (1). Although SA is not the principal lameness diagnosis, it has the highest lameness case fatality rate. In live animals, confirmation of a SA diagnosis relies on arthrocentesis. Synovial fluid collection and pathogen cultivation are considered the gold standard for SA diagnosis; however, positive culture rates for the disease are generally low (1, 2). More rapid tests of the synovial fluid can also lead to the diagnosis and differentiation of SA from non-infectious arthritis. Typically, SA diagnoses are based on the joint fluid having a change in viscosity, an increase in protein levels, and the presence of white blood cells (3, 4). Technological advancements to DNA-sequencing workflows, mass spectrometry, and antigen microarray have been proposed but not extensively evaluated for SA diagnoses (5). Thus, current treatment of SA in cattle relies on empirical medicine based on limited diagnostic and epidemiological data.

*Mycoplasmopsis bovis* (formerly *Mycoplasma bovis*) has been the most frequently reported cause of SA in cattle. Experimental infections via intraarticular or intravenous injections confirmed that *M. bovis* can cause SA (6, 7), and the organism has been identified in septic joints through direct isolation (6–10) and indirect assay (11–13). Additional causative agents and joint-associated bacteria have been identified through cultivation efforts from both beef and dairy animals. These include *Histophilus somni* (14), *Mannheimia haemolytica*, *Trueperella pyogenes* (2, 8), *Streptococcus* spp. (2), *Helcococcus ovis* (15), and *Metamycoplasma alkalescens* (16). Notably, *M. bovis*, *M. haemolytica*, *H. somni*, and *T. pyogenes* also participate in bovine respiratory disease (BRD), which is the most common infectious disease of cattle that is managed by antimicrobial use (AMU). Significantly, most cattle that develop SA do so subsequent to or concurrent with BRD (2, 17). In light of prior or overlapping needs to treat BRD and a relatively small number of reports on pathogens from different food production systems, antimicrobial treatment of SA cases varies between beef and dairy cattle. In feedlot cattle, the parenteral administration of antibiotics that generally demonstrate efficacy against *M. bovis* and *H. somni* alongside nonsteroidal anti-inflammatory drugs (NSAIDs) for pain management is recommended (18). In dairy animals, coagulase-negative streptococci are considered probable causative agents of disease when determining treatment strategies (2). An improved understanding of the presumptive etiological agents of SA and their expected antimicrobial susceptibilities could better inform AMU.

We undertook a survey of the causative agents of SA in western Canadian feedlots by sampling live and dead animals diagnosed with the disease. Microbial community profiles and two distinct metagenomic approaches were employed for pathogen surveillance. In particular, targeted metagenomic analyses by DNA capture sequencing (CapSeq) provided improved pathogen detection and broadened our understanding of clinically relevant antimicrobial resistance (AMR) therein. This combined microbial community and metagenomic analysis highlighted the prevalence of well- and less-appreciated bacterial pathogens that contribute to SA. Simple infections by *M. bovis*, *M. alkalescens*, *T. pyogenes*, and *H. somni* were observed. In the case of *M. bovis* and *M. alkalescens*, isolation, whole-genome sequencing, and phenotypic assays supported cultivation-independent observations of AMR genotypes. In addition to simple infections, mixed infections or complex bacterial communities were found in septic joints. The results call attention to underappreciated and potentially unreported causes of SA that require further study.

## RESULTS AND DISCUSSION

### Overview of sample collection

**Fig 1:**
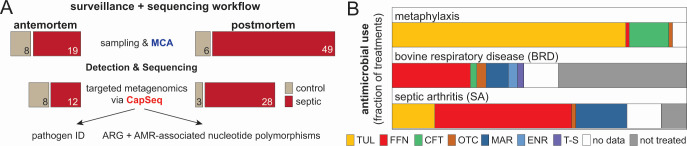
Overview of surveillance and characterization of septic arthritis cases in western Canada. (**A**) Samples and surveillance-based sequencing workflow highlighting the capacity to detect and characterize pathogens and AMR determinants via targeted metagenomics. (**B**) Stacked bar charts summarizing AMU data provided for animals that were part of the study. AMU included tulathromycin (TUL), florfenicol (FFN), ceftiofur (CFT), oxytetracycline (OTC), marbofloxacin (MAR), enrofloxacin (ENR), and trimethoprim-sulfadoxine (T-S).

Ten feedlots in western Canada provided an opportunity to study cattle, resulting in a total of 82 joint samples from 68 SA and 14 control cases (Fig. 1A; Table S1). Antemortem septic and control joints (AMSJ, *n* = 19 and AMCJ, *n* = 8) as well as postmortem septic and control joints (PMSJ, *n* = 49 and PMCJ, *n* = 6) were sampled. In two cases, joint samples were obtained before and after death from the same animals and, in three cases, two infected joints from animals with polyarthritis were sampled. Sampling was conducted in the winter months (December–February) on calves that were placed in the fall. A veterinarian’s diagnosis guided sampling of presumptive septic and control joints from healthy animals or asymptomatic joints of culled individuals. SA can arise from a penetrating injury or as hematogenous infection: animals with grossly identifiable penetrating injuries were excluded from this study. Feedlot treatment histories were provided for most cases, indicating that at least 32 of the 63 animals suffering from SA that were sampled also had a history of BRD (Fig. 1C; Table S1). Tulathromycin (TUL) was the most frequently administered metaphylactic agent (≥80% of animals) to help manage BRD during the study, whereas florfenicol (FFN), oxytetracycline (OTC), ceftiofur, enrofloxacin, marbofloxacin (MAR), or trimethoprim/sulfadoxine were employed as individual treatments for respiratory disease. SA cases were reported to have been treated with FFN, MAR, OTC, or TUL. Data on the use of in-feed antimicrobials (e.g., OTC and tylosin) were not collected but is a common industry practice.

### Simple or complex bacterial community profiles are found in animals suffering from SA

**Fig 2:**
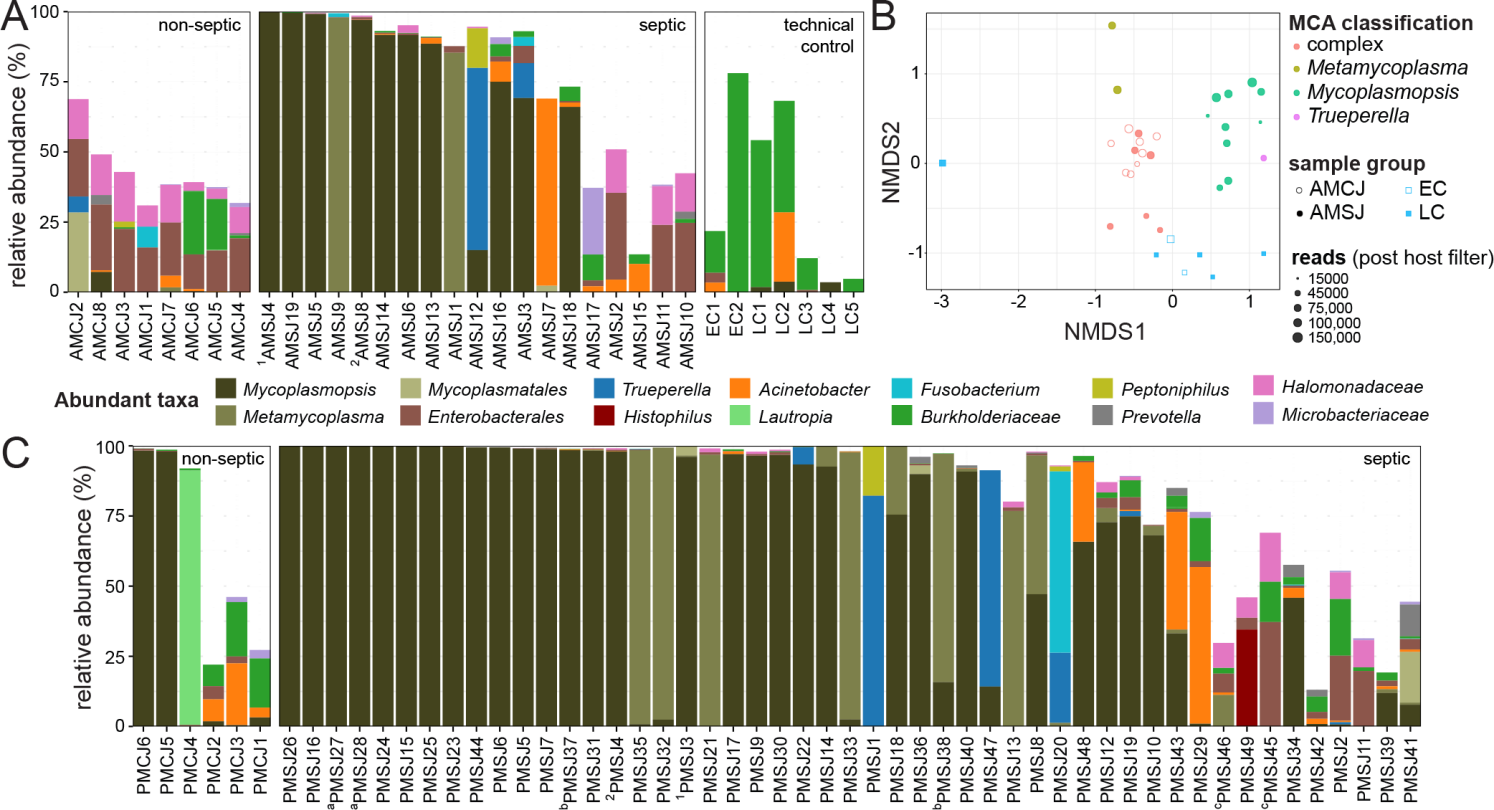
Microbial community analysis from cattle with septic arthritis. (**A**) Bar plots showing abundant members of bacterial communities identified in antemortem control (left; AMCJ), septic joint (middle; AMSJ), and technical control samples (right). (**B**) Ordination plot showing non-metric multidimensional scaling of bacterial communities identified in AMCJ, AMSJ, and control samples. Dots are scaled to post-host filtered read count, colored by MCA classification, and either filled or unfilled to distinguish sample groups. (**C**) Bar plots showing abundant members of bacterial communities identified in postmortem control (left; PMCJ) and septic joints (right; PMSJ). All bar plots are arranged by increasing Shannon index values (Table S2) and show abundant taxonomic groups across all samples as well as known pathogenic groups, including *Histophilus* and *Trueperella*. Superscripts indicate samples taken from the same joint (numbers) or different joints (letters) from the same animal.

To catalog contributors to SA, DNA isolated from septic and control joints was used to identify bacteria by partial 16S rRNA gene amplicon sequencing (Fig. 2). Microbial community analysis (MCA) revealed *Mycoplasmopsis* to be the most frequently SA-associated genus in both antemortem and postmortem septic joints based on relative bacterial abundance (Fig. 2A and C; Table S2). In 31 of 60 *Mycoplasmopsis*-containing samples, the taxon accounted for >90% of the observed bacterial community, including 24 instances in which ≥95% of the reads were assigned to the genus. In two cases, ante- and postmortem sampling was performed from the same joint. AMSJ4 and PMSJ3 were collected from one animal’s joint and AMSJ8 and PMSJ4 from another; the bacterial communities for all four samples were dominated by *Mycoplasmopsis* (Fig. 2). Of the 68 septic joint samples, *Mycoplasmopsis* co-occurred with other known causes of SA: *Metamycoplasma* (18 samples), *Trueperella* (5 samples), and *Histophilus* (1 sample). In other SA cases with limited to undetectable levels of *Mycoplasmopsis*, three genera, *Metamycoplasma*, *Trueperella*, and *Acinetobacter*, were observed as the most abundant taxon. *Acinetobacter* spp. were also detected in technical control samples, suggesting it may be an artifact; however, experimental samples (e.g., AMCJ and AMSJ) differed significantly from each other as well as from extraction and sequencing controls with respect to the compositions of their microbial communities (Fig. 2B). In contrast to diseased joints, the communities observed from healthy animals were more complex, with the exception of two postmortem controls dominated by *Mycoplasmopsis*. While no signs of SA were noted in these two animals, the presence of *Mycoplasmopsis* may reflect hematogenous spread from common sites of their residence, treatments with ceftiofur (a β-lactam to which *Mycoplasmopsis* are intrinsically resistant), and/or postmortem population expansion otherwise controlled in live healthy animal joints. *M. bovis* has also previously been isolated from cattle joints without evidence of arthritis (19). Generally, the survey demonstrated that *Mycoplasmopsis*, presumably *M. bovis*, is the most prevalent cause of SA in western Canadian feedlot cattle and that *Metamycoplasmopsis*, presumably *M. alkalescens*, is an often-overlooked contributor to this disease, appearing in around 20% of SA cases in this study and a recently completed UK-based survey (20). In fact, *Metamycoplasmopsis* accounted for >95% of the assigned taxa in five samples in which the detection of *Mycoplasmopsis* was limited (≤2.5%) or undetected.

Septic joint samples were found to contain a range from simple to complex bacterial communities. Individual α-diversity values, measured using the Shannon diversity index (*H*′), ranged between 0 (e.g., all *Mycoplasmopsis*) and 4.0 for clinical and control samples (Table S2). Notably, the α-diversity values for a subset of five clinical samples (AMSJs; *H*′ = 2.72–3.30) were comparable to those of the eight live control animals (AMCJs; *H*′ = 2.49–4.00). A total of 619 bacterial taxa were identified in the 27 antemortem and 7 technical control samples with the aforementioned subset of complex clinical cases resembling healthy controls (Fig. 2B). Thus, whereas a primary agent or select few could explain disease in 14 of 19 live animals suffering from SA, about a quarter of cases were not simply correlated to known or presumptive disease-causing agents. Misdiagnoses may explain these cases; however, our analysis is blind to the potential contributions of non-bacterial infectious agents that have yet to be studied in the context of beef cattle SA. Finally, an improved or whole joint sampling strategy on postmortem animals might also help to explain these results as organisms associated with the synovial lining, for example, which might be underrepresented or more challenging to detect in the fluid samples. In the future, unbiased microbial isolation efforts may help to reveal and implicate additional causative agents that cannot be reliably predicted by DNA-based methods. To this end, postmortem sampling of two joints from the same afflicted animal revealed similar and distinct microbial communities. In two of three cases, similar communities were observed whether simple (PMSJ27/28) or complex (PMSJ45/46). In a third case, joints differed in that the communities were characterized as being dominated by either *Mycoplasmopsis* or a mix of Metamycoplasma and *Mycoplasmopsis* (PMSJ37/38).

### CapSeq revealed the presence of antimicrobial-resistant *M. bovis* within joints

**Fig 3:**
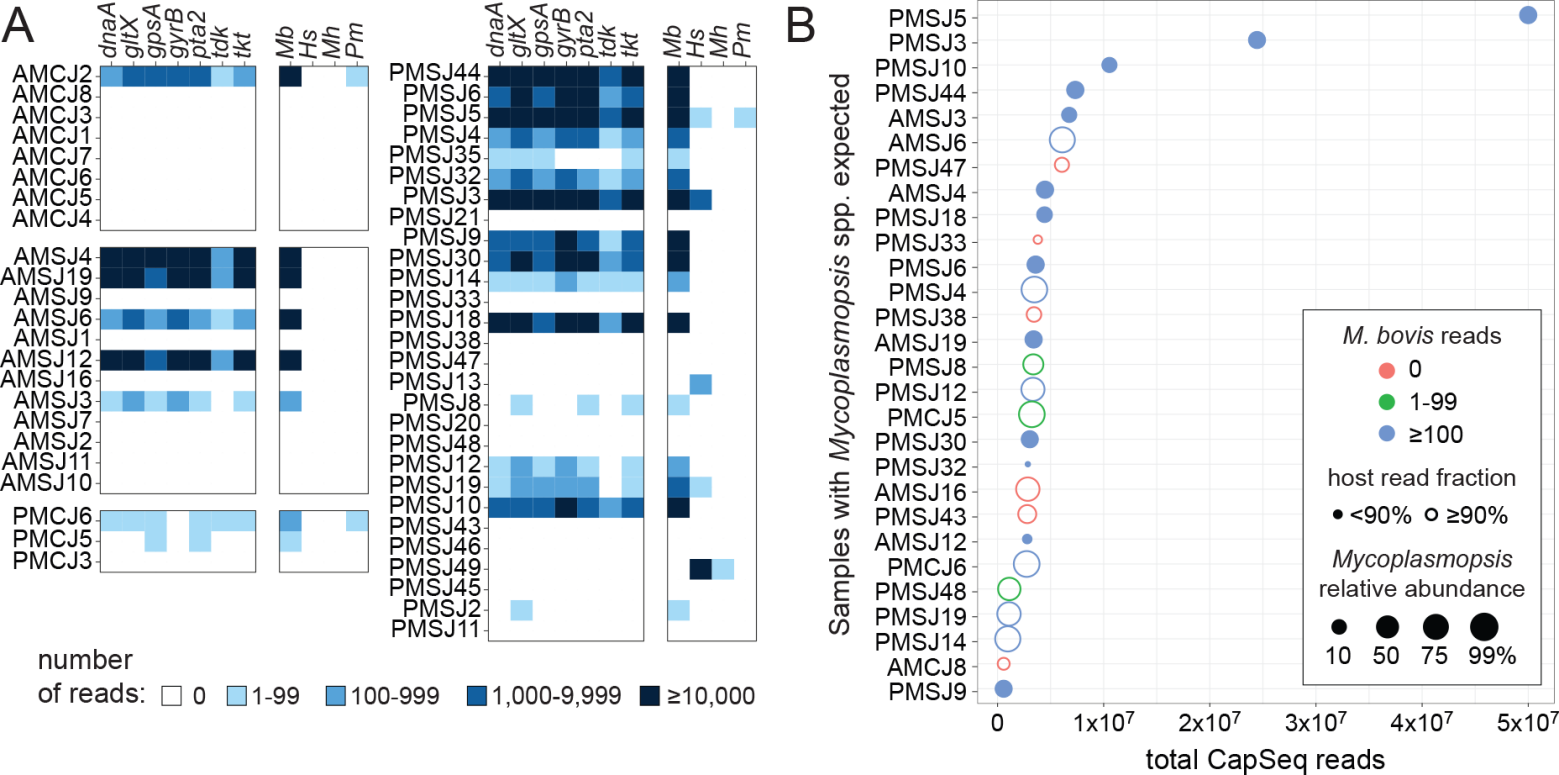
Detection of specific cattle-associated pathogens using CapSeq. (**A**) Heatmap showing the detection for *M. bovis* (*Mb*), *H. somni* (*Hs*), *M. haemolytica* (*Mh*), and *P. multocida* (*Pm*) based on seven-gene MLST schemes. The seven-gene scheme used for *M. bovis* is shown in the left panels with shades of blue coloring indicating numbers of reads associated with each target. The right panels show the combined counts for *M. bovis* as well as those for the other three pathogens’ MLST schemes. (**B**) Dot plot comparing *Mycoplasmopsis* detection via MCA and CapSeq analysis. Samples with *Mycoplasmopsis* spp. are plotted against the total number of CapSeq reads from each sample. The relative abundance (%; only samples ≥1% are shown) from MCA is scaled by dot size and the read fraction attributed to *B. taurus* is depicted using open or closed dots (90% cutoff). Coloring is used to highlight detection of *M. bovis* via CapSeq.

To gain more insight into the nature of the *Mycoplasmopsis* infections, we performed specific DNA target capture and sequencing experiments on a subset of joint samples (*n* = 51). The workflow, which was recently applied to feedlot water and clinical samples, includes seven-gene multilocus sequencing typing (MLST) schemes for *M. bovis*, *H. somni*, *M. haemolytica*, and *Pasteurella multocida* (21). Detection of the targeted *Pasteurellaceae* was sporadic, occurring in only 4 of 55 joints (AMCJ2, PMCJ6, PMSJ5, and PMSJ49; Fig. 3A, right panels). *H. somni* detection in PMSJ49 was expected based on the relative abundance of the genus observed by MCA (34%). In contrast to the dearth of *Pasteurelleceae* in the data set, we expected 28 samples to contain *Mycoplasmopsis* DNA. Presumptive detection of *M. bovis* was possible for only 22 of these 28 samples using CapSeq (Fig. 3A). In the other six (AMCJ8, AMSJ16, PMSJ33, PMSJ38, PMSJ43, and PMSJ47), discordance between the MCA and CapSeq data sets appeared to be multifactorial (Fig. 3B; Table S3). Total read count, relative abundance of *Mycoplasmopsis*, and the host read abundance appear to impact *M. bovis* detection by CapSeq. For example, all samples expected to contain but devoid of *M. bovis* reads by CapSeq were high in bovid DNA content (host read fraction ≥ 90%) and some also possessed relatively low read counts and/or relative abundance of the target genes based on the MCA. In contrast, we also noted the presence of *M. bovis* in an unexpected sample (AMCJ2), explained by misannotation to a close relative of *Mycoplasmopsis* during MCA (Table S2; taxid ID: Eperythrozoon). Overall, the CapSeq data sets not only supported the involvement of *M. bovis* in SA, they also provided a snapshot of prevalent sequence types (STs; Table S3). In general, a single *M. bovis* ST could be identified per joint, including ST2, ST16, ST60, ST65, and ST80. One animal (PMSJ10) appeared to have suffered from a mixed *M. bovis* infection (Table S3).

**Fig 4:**
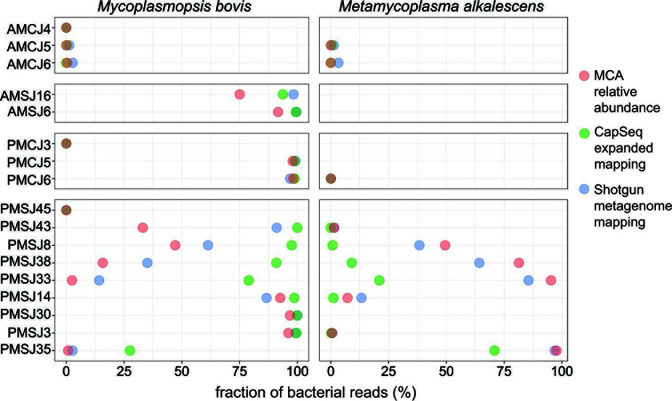
A comparison of *M. bovis* and *M. alkalescens* detection via three different sequencing approaches. Dot plot showing the fraction (%) of *M. bovis* (left) and *M. alkalescens* (right)-assigned reads from microbial community analysis (pink), CapSeq (green), and shotgun metagenomic data sets (blue).

Discordance observed between the MCA and MLST-based CapSeq detection pipeline prompted investigation beyond the MLST targets. An alternative read-mapping strategy to a custom database that included the *B. taurus* genome and genomes of known cattle pathogens was employed. We rationalized that the CapSeq probes, which were designed to enrich for additional *M. bovis* genes (21), would provide greater insight (Table S4). In parallel, we performed conventional shotgun metagenomic sequencing on a subset of samples (*n* = 16) and used the same custom database for *M. bovis* detection. In contrast to the MLST strategy, read mapping to *B. taurus* and a library of full bacterial genome sequences revealed concordance between the data sets and showed that CapSeq outperformed the shotgun approach for *M. bovis* detection (Fig. 4). With the expanded genome mapping approach, *M. bovis* was readily detected in samples characterized by low or no detection using the seven-gene MLST approach (e.g., PMSJ8, PMSJ33, PMSJ35, and AMSJ16). Bacterial reads assigned to *rrs* (16S rRNA) and *rrl* (23S rRNA) loci typically outnumbered assignment to all seven MLST targets combined (Table S3) as well as other specific *M. bovis* target (Table S4). These additional loci also afforded detection of *M. alkalescens* by CapSeq (Fig. 4). Thus, although *M. alkalescens* targets were not included in the experimental design, relatively high sequence identity with *M. bovis* (e.g., 85% for *rrs* and *rrl* genes) was sufficient for capture and provided support for the presence of *M. alkalescens* in septic joints. Finally, it should be noted that the dominance of ribosomal targets in captured sequence space provides a generalizable example of the utility of including multicopy universal target sequences in more specific analyses, but this can come with the negative consequence of diluting capture and sequencing of other informative loci.

**Fig 5:**
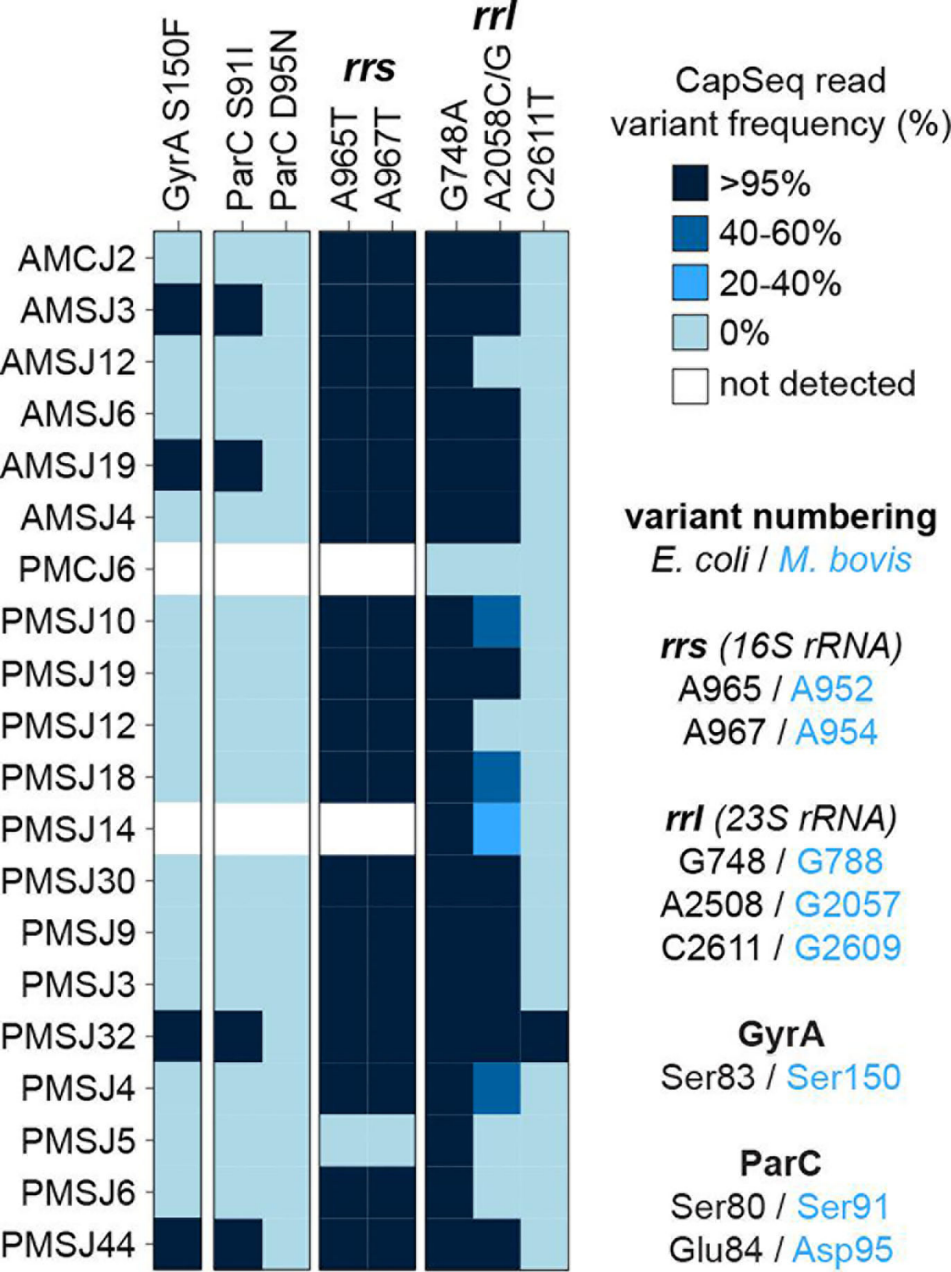
Detection of *M. bovis* determinants of AMR via CapSeq. Heatmap showing genes with polymorphisms associated with AMR in *M. bovis*. Protein variants of GyrA and ParC are shown using *M. bovis* numbering, whereas nucleotide variants in the *rrs* and *rrl* genes are listed following the *E. coli* convention. For clarity, equivalent sites of mutations between *M. bovis* and *E. coli* are listed at the right.

To exploit sequencing beyond identification and typing, the *M. bovis* CapSeq probes were designed to investigate nucleotide polymorphisms implicated in AMR (21). *M. bovis* typically evades the action of antibiotics through adaptive mutation to their targets. This includes mutations to the *rrs* and *rrl* genes as well as mutations in quinolone resistance-determining regions (QRDRs) of gyrase (e.g., *gyrA* and *gyrB*) and topoisomerase (e.g., *parC* and *parE*) genes (22, 23). Indeed, CapSeq afforded the identification of a series of nucleotide polymorphisms in *M. bovis rrs*, *rrl*, *gyrA*, and *parC* genes (Fig. 5). Specifically, the majority of *rrs* sequences contained A965T and A967T mutations (*Escherichia coli* numbering convention), which is correlated to reduced susceptibility to tetracyclines in *M. bovis* (24). All *M. bovis rrl* sequences contained the G748A polymorphism, which confers resistance to macrolides, and the majority of sequences also contained at least one other nucleotide polymorphism, A2057G/C or C2611T, associated with macrolide resistance (25–27). In four instances, co-occurrent mutations to the QRDR encoded within *gyrA* and *parC* (S83F and S80I; *E. coli* numbering) were observed. Accordingly, all SA-associated *M. bovis* are predicted to be macrolide resistant, less susceptible to the action of tetracyclines, and a subset with GyrA and ParC variants is predicted to be resistant to fluoroquinolones. The observations of these genotypes are in line with known AMU: macrolides and tetracyclines have long served as conventional treatments and cattle feed additives, whereas fluoroquinolones (e.g., enrofloxacin and marbofloxacin) are employed to explicitly treat disease (Table S1).

### CapSeq suggests the presence of AMR *T. pyogenes* in septic joints

**Fig 6:**
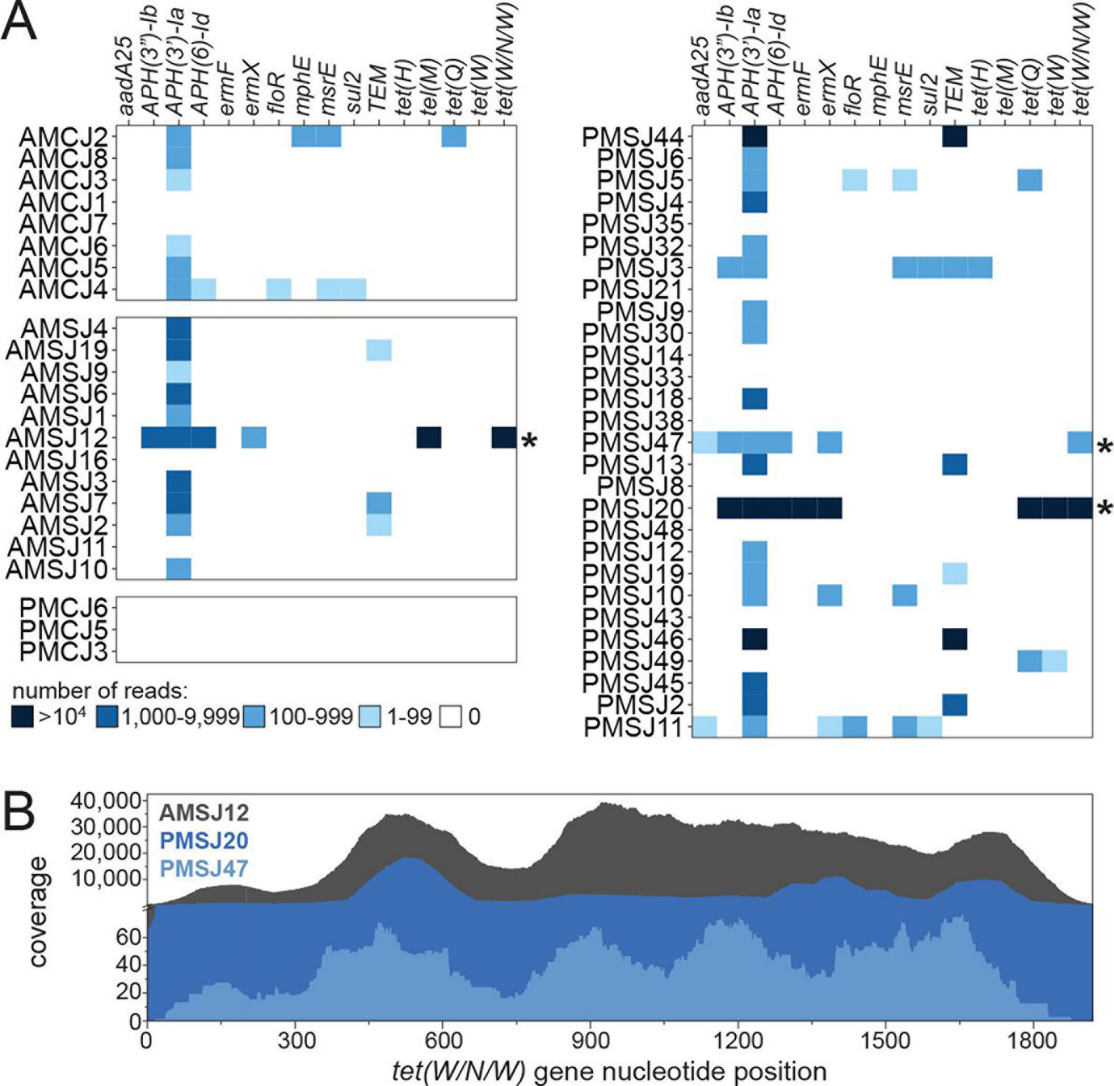
Detection of ARGs by CapSeq. (**A**) A focused summary of the results of ARG detection in joint DNA samples highlighting clinically relevant and commonly observed genes in the feedlot environment. Asterisks denote the detection of a *T. pyogenes*-specific ARG. (**B**) Mapping of CapSeq reads across the *tet(W/N/W*) gene observed in samples AMSJ12, PMSJ20, and PMSJ47.

The CapSeq panel included probes for additional ARGs known to be encoded by other cattle pathogens. Overall, detection of these ARGs was relatively limited (Fig. 6A), consistent with the prevalence and abundance of organisms known to lack them (*M. bovis and M. alkalescens*). For example, the *floR* gene, which encodes for a FFN efflux pump, was detected in only three joint samples despite FFN use by at least 8 of the 10 feedlots during the study (Table S1). Nevertheless, CapSeq detection of ARGs from this clinical material greatly outperformed a conventional shotgun metagenomic approach, which failed at reliable detection of resistance determinants (Table S5). Accordingly, this approach could be implemented to better address pathogen and AMR detection in future investigations of agricultural systems, matching similar efforts in human medicine (28, 29).

The utility of the CapSeq approach was highlighted by specific ARG detection suggesting the presence of OTC-resistant *T. pyogenes* in septic joints. Coincidental detection of *tet(W/N/W*) by CapSeq and *T. pyogenes* by MCA was remarkable. The *tet(W/N/W*) gene is nearly confined to *T. pyogenes* and often co-occurs with *ermX* (30–32) as observed in the SA joint samples (Fig. 6A), and was detected in three samples expected to possess the organism via MCA: AMSJ12, PMSJ20, and PMSJ47 (Fig. 6B). Few other bacterial species encode for the *tet(W/N/W*) gene (based on nucleotide sequence identity >98%), though many related ribosomal protection protein-encoding genes exist (e.g., *tetW*). Consensus nucleotide sequences for reads mapped to *tet(W/N/W*) from AMSJ12 and PMSJ47 data sets were identical to the reference sequence (GenBank: AY049983.2, 8,309…10,228) whereas that constructed for PMSJ20 was 98.9% identical. Thus, the sequence identity of the *tet(W/N/W*) reads observed in the joint samples suggested that they originated from *T. pyogenes*. The identification of tetracycline-resistance gene encoding sequences is consistent with a recent report that ~60% *T. pyogenes* isolated from cattle carry the *tet(W/N/W*) gene (30) as well as the in-feed delivery of OTC and its specific use for SA treatments.

### Cultivation from SA joints corroborates detection of antimicrobial-resistant bacteria

To complement the DNA-based detection of AMR, *M. bovis* were isolated from a subset of control and septic joints. A total of 11 *M*. *bovis* isolates were subjected to antimicrobial susceptibility tests (ASTs; Table 1), 5 of which were characterized using whole-genome sequencing (Table 1; Table S6). We focused on *M. bovis* since it was the major pathogen found in SA cases and can be readily isolated in the laboratory through the use of selective media. A panel of feedlot relevant antibiotics was used to determine minimum inhibitory concentrations (MICs) in pleuropneumonia-like organism (PPLO) broth. All joint isolates were determined to be resistant to both 15- and 16-atom-containing macrolides (tulathromycin, gamithromycin, tylosin, and tildipirosin; MICs > 256 μg/mL) whereas only a subset demonstrated elevated MICs for other classes of antibiotics. Overall, the ranges of measured MICs were similar to previous reports. MIC_50_ values for ENR, FFN, and tetracyclines, for example, were reported as 0.25, 2, and 2 μg/mL, respectively, from isolates originating from Germany, Canada, and Australia (33–35). In particular, *M. bovis* from PMSJ14, PMSJ48, and PMCJ5 was capable of growing in the presence of 4–8 μg/mL ENR. This phenotype was explained by GyrA S150F and ParC S91I or D95N variants in the QRDR (*M. bovis* numbering). MICs ≥4 μg/mL were also observed for FFN and OTC; whereas reduced susceptibility to FFN is not well understood at a molecular level in *M. bovis*, OTC resistance can be explained by the *rrs* A952T and A954T mutations (*E. coli* numbering). In most cases, the AMR genotypes were also observed in CapSeq data; however, we noted that isolates from a control joint, *M. bovis* PMCJ5 and PMCJ5-2, belonged to previously unreported STs (ST293 and ST295; Table S6) and carried mutations that were not dominant in CapSeq data sets (e.g., *rrl* C2611G and ParC D95N). The differences in *M. bovis* abilities to be cultivated in the laboratory may or may not reflect their true abundance in septic joints. Furthermore, many clinical joint samples were heterogeneous in nature, showing signs of inflammation and requiring physical disruption (e.g., mincing with sterile scissors thereby dividing the material) during sample preparation for DNA and cultivation, which might explain differences in cultivation-dependent and independent methods.

**TABLE 1:** MICs determined for *M. bovis* and *M. alkalescens* isolates from septic joints^*a*^

Isolate	MIC (μg/mL) of:				AMR genotype^*c*^	ST	GenBank no.
	ENR	FFN	OTC	Macrolides^*b*^			
*M. bovis*							
AMSJ16	≤0.25	2	2	>256	*rrl1/2*: G748A, *rrl1* A2058G, *rrl2* C2611G, *rrs*: A965T, A967T	239	CP194394.1
AMSJ16-2	≤0.25	2	4	>256			
AMSJ16-3	≤0.25	4	4	>256			
AMSJ16-4	≤0.25	2	2	>256			
AMSJ16-5	≤0.25	4	4	>256			
PMSJ14	≤0.25	4	4	>256	*rrl1/2*: G748A, *rrl1* A2058G, *rrl2* C2611G, *rrs*: A965T, A967T	239	CP194392.1
PMSJ14-2	≤0.25	4	4	>256			
PMCJ5	4	8	4	>256	*rrl1/2*: G748A, A2058G, *rrs*: A965T, A967T, GyrA S150F, ParC S91I	293	CP190343.1
PMCJ5-2	4	16	4	>256	*rrl1/2*: G748A, *rrl1* G2059, *rrs*: A965T, A967T, GyrA S150F, ParC D95N	295	CP194393.1
PMSJ48	8	4	≤2	>256	*rrl1/2*: G748A, A2058A, *rrs*: A965T, A967T, GyrA S150F, ParC S91I	60	CP194391.1
PMSJ48-2	8	4	≤2	>256			
*M. alkalescens*							
PMSJ35	≤0.25	8	≤2	>256	*rrl1/2*: G748A, *rrs1* A965G		CP190015.1

^*a*^ All were resistant to ceftiofur (>512 μg/mL; not shown).

^*b*^ Macrolides included tulathromycin, gamithromycin, tylosin, tilmicosin, and tildipirosin.

^*c*^ *E. coli* numbering is used for *rrl* (23S rRNA) and *rrs* (16S rRNA); *M. bovis* numbering is used for GyrA and ParC (for comparison of positional equivalent mutations see Fig. 5).

**Fig 7:**
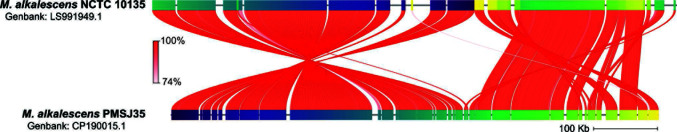
Comparison of *M. alkalescens* genomes. Linearized *M. alkalescens* genome maps showing 233 orthologous matching segments used to determine ANI between the PMSJ35 isolate and type strain.

In addition to *M. bovis*, *M. alkalescens* has been reported to cause SA. Isolation of *M. alkalescens* from symptomatic dairy cattle and intraarticular, but not intravenous, inoculations of healthy animals demonstrated it as the cause of SA nearly five decades ago (16). In this first report, which documented ~30 SA cases in a herd of 215 animals, the disease was suspected to be linked to umbilical exposure with *M. alkalescens*-induced omphaloarteritis preceding SA. Additional reports have implicated *M. alkalescens* as a cause of SA (20, 36), respiratory disease (37), mastitis (38), and abortion (39) in cattle. Here, *Metamycoplasma* was the second most abundant SA-associated genus, and we isolated *M. alkalencens* from a sample expected to be enriched in the genus (PMSJ35; Fig. 1B; 98% relative abundance). MICs for the PMSJ35 isolate were within the reported ranges for other cattle-associated *M. alkalescens* (40, 41). Similar to *M. bovis*, *M. alkalescens* PMSJ35 was resistant to macrolides, and whole-genome sequencing revealed that it contained the *rrl* G748A polymorphism correlated to macrolide resistance. The isolate was also characterized by phenotypic sensitivities to OTC and ENR while showing reduced susceptibility to FFN. Notably, there are only two other reported *M. alkalescens* genomes currently available: a closed genome for the type strain NCTC 10135 (ATCC 2910), which was isolated from a nasal cavity (42) and a draft genome of *M. alkalescens* 14918, which was isolated from lung tissue from a calf that suffered from pneumonia (43). The *M. alkalescens* PMSJ35 closed circular 767,707 bp genome is ~57 kb smaller than the NCTC 10135 sequence with which it shares 98.5% average nucleotide identity (ANI; Fig. 7). Future studies should focus on *M. alkalescens* isolation and characterization considering the relatively limited information that is available for this species, which is frequently present in SA cases.

### Improving and implementing surveys and characterizations of SA in cattle

This DNA-focused survey of causative agents of SA in feedlot cattle confirmed the prominent role of *M. bovis* and revealed the involvement of underappreciated taxa in this seriously debilitating disease. Importantly, the identification of *M. alkalescens* in samples taken from seven different feedlots implicates it as a common contributor to SA in western Canada (Table S1). Moreover, *T. pyogenes*, including AMR genotypes, were identified more frequently and at higher relative abundance than *H. somni*, which is anecdotally thought of as a prominent cause of SA in the region. Finally, *Acinetobacter* spp. was routinely detected in SA samples, including as the dominant taxon in AMSJ7, co-infecting with *M. bovis* in PMSJ48, and as a member of more complex communities like PMSJ15 (Fig. 2; Table S2). Whether or not *Acinetobacter* spp. contribute directly to disease remains to be determined. More broadly, how complex communities that lack known causative agents of SA generate inflammation remains a significant epidemiological and molecular question. Ultimately, a better understanding of SA cases is likely to help guide the empirical medicine that is used to treat animals suffering from this disease.

## MATERIALS AND METHODS

### Animals, case selection, and sampling

All participating feedlots were typical of the western Canadian industry, receiving diets formulated to meet or exceed National Research Council standards (44), and included monensin, an ionophore, at 22–33 ppm based on dry matter. AMU to manage disease, including BRD and SA, occurred in treatment facilities (hospital pens) where veterinarians confirmed the case definitions and enrolled animals for sampling. The treatment histories of animals, collection year, and additional metadata (e.g., anonymized feedlot identifiers) were recorded when possible (Table S1). Samples collected in 2021 were immediately mixed 1:1 with RNAlater, whereas those collected in 2022 were not. All samples were frozen at −20°C at a veterinary clinic before transportation in the frozen state to a research laboratory, where they were stored at −80°C until further processing.

Live animals were sampled by arthrocentesis after a 15 cm^2^ area was clipped and scrubbed with 4% chlorhexidine gluconate and 70% rubbing alcohol solutions. Animals were also provided with a local anesthetic, lidocaine, before a veterinarian used a 3.8 cm needle to sample the joint capsule. Synovial fluid was aspirated into a syringe before animals were administered an oral dose of meloxicam, an NSAID, at 1 mg/kg BW. While a 14- or 16-gage needle was required for animals suffering from SA, fluid samples from healthy joints were retrieved using 18- and 20-gage needles. For the presumptive SA cases, animals had at least one swollen joint, no signs of lesions, and no previous history of lameness. Control animals were selected from the same pens as infected animals.

Dead animal samples, cases, and controls were identified by evening postmortem exams conducted by a feedlot veterinarian. SA cases were grossly identified by joint swelling, whereas postmortem control cases did not show joint swelling but succumbed to another illness. A clean knife was used to reflect the skin from the joint with manual traction applied to reduce contact and potential contamination of the subcutaneous tissue over the joint. A 14- or 16-gage needle was then inserted into the joint capsule and synovial fluid aspirated into a syringe. All cases were samples <24 h from time of death. All animals, live or dead, were fall-placed calves sampled in early winter.

### Extraction of DNA from joint samples

DNA was extracted from joint fluid samples using two protocols: a phenol:chloroform extraction of sedimented materials or a Qiagen Blood & Tissue kit. Samples, in particular those from SA cases, were cut up using sterile surgical scissors in an aseptic environment. These minced samples were weighed to ~25 mg to comply with kit specification before extraction and pooling as necessary to meet yield requirements for DNA sequencing experiments. In nearly all instances, DNA obtained by phenol:chloroform extraction was re-purified using an AMPure XP magnetic bead kit (Beckman Coulter Inc, CA, USA). Technical controls were performed alongside DNA extractions and sequencing library preparations (see below) using common kits and reagents.

### Microbial community profiling

PCRs (30 cycles) were performed with Q5 High-Fidelity 2X Master Mix (New England Biolabs, MA, USA) containing 2 ng DNA and 1 μM 515F/806R 16S V4 rRNA primers as previously described (45). Amplicon library clean-up was completed using AMPureXP magnetic beads. An index PCR (10 cycles) was performed with Q5 High-Fidelity 2X Master Mix and i5 and i7 indices following the Nextera XT Index Kit v2 (Illumina, San Diego, CA, USA) specification. Indexed libraries, purified using AMPureXP magnetic beads, were pooled and size-selected for a ~440 bp band using an E-Gel Size Select 2% gel (Invitrogen, CA, USA). The size-selected library was quantified with the NEBNext Library Quant Kit for Illumina (New England Biolabs) and diluted to 1.6 pM for sequencing. Paired-end (2 × 150 bp) sequences were generated on either an Illumina NovaSeq 6000 or Miniseq platform (300 cycles).

Sequencing reads that mapped to a *Bos taurus* reference genome (ARS-UCD2.0; GCF_002263795.3) were removed from data sets using a local alignment with Bowtie2 (v2.5.1; --no-mixed --no-discordant [46]). TrimGalore (v0.6.10) was utilized with the Nextera and polyA tags to remove adaptors (47) from the reads before they were imported into Qiime2 (Amplicon, v2024.5) and additional filtering of reads with homopolymeric sequences >8 bp was removed using rescript cull-seqs (48, 49). Amplicon sequence variants were generated with DADA2 with minimum overlap set to 7 bp (50), and classified using the Greengenes2 (v2024.09) database (51). Qiime2 results were imported into R using qiime2R (52). After calculating proportional abundance through dividing abundance of taxa by the sum abundance within each sample, Shannon diversity index (*H*) was calculated with the formula *H* = −∑(proportion × ln(proportion)). Genera were considered detected when they made up ≥1% of the total abundance classified.

### Metagenomic sequencing

CapSeq sequencing libraries with a DNA probe set targeting BRD pathogens were prepared as previously described (21), following technical specifications provided by the DNA probe manufacturer (Twist Biosciences, San Francisco, CA, USA). Briefly, following library preparations and quantification, the libraries were pooled for hybridization with a Twist kit (Twist Biosciences, 104180) with the following thermal cycling conditions: 95°C for 5 min and 60°C for 30 min, and post-hybridization PCR amplification was performed after which they were cleaned. CapSeq libraries underwent pair-ended (2 × 150 bp) sequencing on an Illumina NovaSeq6000 platform. DNA libraries for shotgun sequencing were prepared using TruSeq DNA PCR-Free Kit (Illumina) followed by pair-ended (2 × 150 bp) sequencing on one lane of a NovaSeqX Series 10B flow cell.

### Gene detection from metagenomic data sets

Initial read processing for both the CapSeq data (n = 51) and the shotgun metagenomic (n = 17) data sets started with using fastp to (v0.23.4) (53) trim illumina adaptors with a minimum output length of 50 bp. Bowtie2 was used to remove host reads as described above for the 16S rDNA amplified data set. Reads were classified with Kraken2 (2.1.3) (54) with a NCBI RefSeq database constructed out of available complete genomes for *B. taurus*, *M. bovis*, *M. alkalescens*, *M. haemolytica*, *P. multocida*, *H. somni*, *B. trehalosi*, *T. pyogenes*, *M. wenyonii*, and *U. diversum* (data fetched May 2025). MLST scheme genes (55) and genes with AMR associated polymorphisms for *M. bovis*, *M. haemolytica*, *P. multocida*, and *H. somni* were used as references for mapping with processed sequencing data using BBMap (BBTools v39.08; local alignment, 95% identity, kiflter = 25, subflter = 20, maxindel = 3, insfilter = 0, and delfilter = 0) (56). Consensus MLST gene sequences (cutoffs: >95% conservation across all positions, ≥100 mapped reads) were queried using the PubMLST sequence typing tool (pubmlst.org) to identify *M. bovis* STs.

For ARG detection from CapSeq data sets, the resistance gene identifier (RGI, bwt, v6.0.3, bowtie2 aligner) from the Comprehensive Antibiotic Resistance Database (CARD, v4.0.0) was employed (57). Positive ARG detection was considered if ≥100 mapped reads/gene was observed along with ≥80% coverage. Shotgun metagenomic data were analyzed using the RGI tool and a more specific search for selected genes (see Table S5) was performed using BBSplit (BBTools v39.08) (56) as described above. To detect and quantify nucleotide polymorphisms in targeted ARGs, only data sets that included ≥100 reads at the genes of interest were considered.

### Isolation of *M. bovis* and *M. alkalescens*

The isolations of *M. bovis* and *M. alkalescens* were guided by results of the 16S rRNA community profiling. A 20 μL volume of a joint fluid from samples showing “simple” infection types was used to inoculate 2 mL of PPLO broth enriched with 10% yeast extract (wt/vol), 10% horse serum (vol/vol), penicillin G (10,000 IU), and thallium acetate (0.0005% wt/vol; abbreviated hereafter as PPLO broth/agar). The inoculated broth was incubated at 37°C in a humidified 5% CO_2_ environment for 7 days. Then, a 50 μL aliquot was plated on PPLO agar and incubated at 37°C in a humidified 5% CO_2_ environment for 3–5 days before individual colonies were selected for further propagation and cryopreservation.

### DNA isolation, sequencing, and analysis of *M. bovis* and *M. alkalescens* genomes

A PureLink Genomic DNA Mini Kit (Invitrogen) was employed to isolate DNA from 5-day-old cultures. DNA sequencing was performed using Oxford Nanopore Technologies by Plasmidsaurus Inc. (South San Francisco, CA, USA). Amplification-free long-read sequencing libraries were constructed using v14 library chemistry and then sequenced using an R10.4.1 flow cell. High-quality 45–125× coverage genomes were assembled by downsampling data sets. Briefly, read removal was performed by Filtlong v0.2.1 (58) and preliminary assemblies were constructed using Miniasm v0.3 (59) before further downsampling, weighting against low-quality reads. Flye v2.9.1 (60) and Medaka v1.8.0 (61) were then used to generate the final, polished assemblies. Nucleotide polymorphisms in AMR determining genes were identified by sequence alignment and manual inspection. Pairwise genome comparisons of *M. alkalescens* were performed using FastANI (62) within the Proksee platform (63).

### Determination of antimicrobial susceptibilities

Antimicrobial susceptibility testing was performed using PPLO broth amended with alamarBlue (ThermoFisher) at 37°C in a humidified 5% CO_2_ environment. For *M. alkalescens*, the PPLO broth was supplemented with 21.25 mg/L pyruvate. Bacteria were prepared at 10^3^ CFU/mL from single-use glycerol stocks of known viable *M. bovis* or *M. alkalescens* counts and arrayed into 200 μL volumes in 96-well microtiter plates containing twofold dilution series of ENR, FFN, OTC, GAM, TUL, TYL, TIL, and TIP. MICs were assigned to conditions in which no bacterial growth was detected using alamarBlue (ThermoFisher) fluorescence as a proxy. Three independent measurements were performed for each strain. Positive (no antibiotic) and negative (no bacteria) controls were performed in parallel.

### Data visualization

Plots were routinely generated using R, Microsoft Excel, Origin v2024b, and then re-organized and re-colored using Adobe Illustrator to generate multi-panel figures.

## Data Availability

All DNA sequences have been made available through the National Center for Biotechnology Information. BioProjects for microbial community profiles (PRJNA1280048), CapSeq data (PRJNA1140205), and metagenomic data (PRJNA1279254) contain links to the associated data and Sequence Read Archive submissions. Individual accession numbers are listed in Table S1. Whole genomes of *M. bovis* (PRJNA1125005) and *M. alkalescens* (PRJNA1257941) are also available with GenBank accession numbers listed in Table S6.

## SUPPLEMENTAL MATERIAL

Supplemental tables aem.01675-25-s0001.xlsx Tables S1 to S6.

ASM does not own the copyrights to Supplemental Material that may be linked to, or accessed through, an article. The authors have granted ASM a non-exclusive, world-wide license to publish the Supplemental Material files. Please contact the corresponding author directly for reuse.

## References

[1] Desrochers A, Francoz D. 2014. Clinical management of septic arthritis in cattle. Vet Clin North Am Food Anim Pract 30:177–203, doi:10.1016/j.cvfa.2013.11.006 24534665

[2] Constant C, Nichols S, Desrochers A, Babkine M, Fecteau G, Lardé H, Fairbrother J-H, Francoz D. 2018. Clinical findings and diagnostic test results for calves with septic arthritis: 64 cases (2009–2014). J Am Vet Med Assoc 252:995–1005. doi:10.2460/javma.252.8.995 29595396

[3] Rohde C, Anderson DE, Desrochers A, St-Jean G, Hull BL, Rings DM. 2000. Synovial fluid analysis in cattle: a review of 130 cases. Vet Surg 29:341–346. doi:10.1053/jvet.2000.5605 10917284

[4] Weaver AD. 1972. Disease of the bovine stifle joint. Bov pract 7:41–45. doi:10.21423/bovine-vol1972no7p41-45

[5] Pearson GB, Ysebaert MP, Papa B, Reesink HL. 2023. Synovial sepsis diagnostics and antimicrobial resistance: a one-health perspective. J Am Vet Med Assoc 261:1115–1120. doi:10.2460/javma.23.05.0227 37380157

[6] Stalheim OH, Stone SS. 1975. Isolation and identification of Mycoplasma agalactiae subsp. bovis from arthritic cattle in Iowa and Nebraska. J Clin Microbiol 2:169–172. doi:10.1128/jcm.2.3.169-172.1975 1176624 PMC274165

[7] Stalheim OH, Page LA. 1975. Naturally occurring and experimentally induced mycoplasmal arthritis of cattle. J Clin Microbiol 2:165–168. doi:10.1128/jcm.2.3.165-168.1975 1176623 PMC274164

[8] Anholt RM, Klima C, Allan N, Matheson-Bird H, Schatz C, Ajitkumar P, Otto SJ, Peters D, Schmid K, Olson M, McAllister T, Ralston B. 2017. Antimicrobial susceptibility of bacteria that cause bovine respiratory disease complex in Alberta, Canada. Front Vet Sci 4:207. doi:10.3389/fvets.2017.00207 29255716 PMC5723070

[9] Cantón G, Llada I, Margineda C, Urtizbiría F, Fanti S, Scioli V, Fiorentino MA, Louge Uriarte E, Morrell E, Sticotti E, Tamiozzo P. 2022. Mycoplasma bovis-pneumonia and polyarthritis in feedlot calves in Argentina: first local isolation. Rev Argent Microbiol 54:299–304. doi:10.1016/j.ram.2022.02.005 35606271

[10] Shitamori F, Uemura R, Kanda T, Sueyoshi M. 2022. The presence of adhesion factors NOX, α-enolase, TrmFO, P27, and VpmaX in Mycoplasma bovis wild isolates in Japan. Open Vet J 12:782–786. doi:10.5455/OVJ.2022.v12.i6.1 36650870 PMC9805775

[11] Haines DM, Martin KM, Clark EG, Jim GK, Janzen ED. 2001. The immunohistochemical detection of Mycoplasma bovis and bovine viral diarrhea virus in tissues of feedlot cattle with chronic, unresponsive respiratory disease and/or arthritis. Can Vet J 42:857–860. 11708203 PMC1476660

[12] Zhao G, Hou P, Huan Y, He C, Wang H, He H. 2018. Development of a recombinase polymerase amplification combined with a lateral flow dipstick assay for rapid detection of the Mycoplasma bovis. BMC Vet Res 14:412. doi:10.1186/s12917-018-1703-x 30572884 PMC6302395

[13] Adegboye DS, Halbur PG, Nutsch RG, Kadlec RG, Rosenbusch RF. 1996. Mycoplasma bovis-associated pneumonia and arthritis complicated with pyogranulomatous tenosynovitis in calves. J Am Vet Med Assoc 209:647–649. 8755989

[14] Van Donkersgoed J, Janzen ED, Harland RJ. 1990. Epidemiological features of calf mortality due to hemophilosis in a large feedlot. Can Vet J 31:821–825. 17423705 PMC1480899

[15] Jost A, Sickinger M. 2021. Helcococcus ovis associated with septic arthritis and bursitis in calves - a case report. BMC Vet Res 17:291. doi:10.1186/s12917-021-02996-6 34479562 PMC8414772

[16] Bennett RH, Jasper DE. 1978. Mycoplasma alkalescens-induced arthritis in dairy calves. J Am Vet Med Assoc 172:484–488. 624670

[17] Davis-Unger J, Schwartzkopf-Genswein KSG, Pajor EA, Hendrick S, Marti S, Dorin C, Orsel K. 2019. Prevalence and lameness-associated risk factors in Alberta feedlot cattle. Transl Anim Sci 3:595–606. doi:10.1093/tas/txz008 32704830 PMC7200549

[18] Apley MD. 2020. Diagnosis and therapy of feedlot lameness, p 54–59. American Association of Bovine Practitioners.

[19] Gagea MI, Bateman KG, Shanahan RA, van Dreumel T, McEwen BJ, Carman S, Archambault M, Caswell JL. 2006. Naturally occurring Mycoplasma bovis-associated pneumonia and polyarthritis in feedlot beef calves. J Vet Diagn Invest 18:29–40. doi:10.1177/104063870601800105 16566255

[20] Deeney AS, Collins R, Ridley AM. 2021. Identification of Mycoplasma species and related organisms from ruminants in England and Wales during 2005-2019. BMC Vet Res 17:325. doi:10.1186/s12917-021-03037-y 34641885 PMC8513359

[21] Russell JN, Kos D, Yacoub E, Sies AN, Warr B, Jelinski M, Ruzzini A, Cameron ADS. 2025. Enhanced metagenomic surveillance for bovine respiratory disease pathogens and antimicrobial resistance by hybridization capture sequencing. Appl Environ Microbiol 91:e0097725. doi:10.1128/aem.00977-25 40928217 PMC12542742

[22] Gautier-Bouchardon AV. 2018. Antimicrobial resistance in Mycoplasma spp. Microbiol Spectr 6. doi:10.1128/microbiolspec.arba-0030-2018 PMC11633602 30003864

[23] Vester B, Douthwaite S. 2001. Macrolide resistance conferred by base substitutions in 23S rRNA. Antimicrob Agents Chemother 45:1–12. doi:10.1128/AAC.45.1.1-12.2001 11120937 PMC90232

[24] Amram E, Mikula I, Schnee C, Ayling RD, Nicholas RAJ, Rosales RS, Harrus S, Lysnyansky I. 2015. 16S rRNA gene mutations associated with decreased susceptibility to tetracycline in Mycoplasma bovis. Antimicrob Agents Chemother 59:796–802. doi:10.1128/AAC.03876-14 25403668 PMC4335885

[25] Lerner U, Amram E, Ayling RD, Mikula I, Gerchman I, Harrus S, Teff D, Yogev D, Lysnyansky I. 2014. Acquired resistance to the 16-membered macrolides tylosin and tilmicosin by Mycoplasma bovis. Vet Microbiol 168:365–371. doi:10.1016/j.vetmic.2013.11.033 24393633

[26] Sulyok KM, Kreizinger Z, Wehmann E, Lysnyansky I, Bányai K, Marton S, Jerzsele Á, Rónai Z, Turcsányi I, Makrai L, Jánosi S, Nagy SÁ, Gyuranecz M. 2017. Mutations associated with decreased susceptibility to seven antimicrobial families in field and laboratory-derived Mycoplasma bovis strains. Antimicrob Agents Chemother 61:e01983-16. doi:10.1128/AAC.01983-16 27895010 PMC5278709

[27] Prats-van der Ham M, Tatay-Dualde J, de la Fe C, Paterna A, Sánchez A, Corrales JC, Contreras A, Gómez-Martín Á. 2017. Molecular resistance mechanisms of Mycoplasma agalactiae to macrolides and lincomycin. Vet Microbiol 211:135–140. doi:10.1016/j.vetmic.2017.10.012 29102109

[28] Guitor AK, Katyukhina A, Mokomane M, Lechiile K, Goldfarb DM, Wright GD, McArthur AG, Pernica JM. 2024. Minimal impact on the resistome of children in Botswana after azithromycin treatment for acute severe diarrheal disease. J Infect Dis 230:239–249. doi:10.1093/infdis/jiae049 39052715 PMC11272098

[29] Hackenberger D, Imtiaz H, Raphenya AR, Alcock BP, Poinar HN, Wright GD, McArthur AG. 2025. CARPDM: cost-effective antibiotic resistome profiling of metagenomic samples using targeted enrichment. Appl Environ Microbiol 91:e0187624. doi:10.1128/aem.01876-24 40019273 PMC11921354

[30] Magossi G, Gzyl KE, Holman DB, Nagaraja TG, Amachawadi R, Amat S. 2025. Genomic and metabolic characterization of Trueperella pyogenes isolated from domestic and wild animals. Appl Environ Microbiol 91:e0172524. doi:10.1128/aem.01725-24 39745423 PMC11784230

[31] Marchionatti E, Kittl S, Sendi P, Perreten V. 2024. Whole genome-based antimicrobial resistance, virulence, and phylogenetic characteristics of Trueperella pyogenes clinical isolates from humans and animals. Vet Microbiol 294:110102. doi:10.1016/j.vetmic.2024.110102 38749210

[32] Kos D, Jelinski M, Ruzzini A. 2025. Retrospective analysis of antimicrobial resistance associated with bovine respiratory disease. Appl Environ Microbiol 91:e0190924. doi:10.1128/aem.01909-24 39918326 PMC11921372

[33] Gütgemann F, Müller A, Churin Y, Kumm F, Braun AS, Yue M, Eisenberg T, Entorf M, Peters T, Kehrenberg C. 2023. Toward a method for harmonized susceptibility testing of Mycoplasma bovis by broth microdilution. J Clin Microbiol 61:e0190522. doi:10.1128/jcm.01905-22 37439667 PMC10446863

[34] Jelinski M, Kinnear A, Gesy K, Andrés-Lasheras S, Zaheer R, Weese S, McAllister TA. 2020. Antimicrobial sensitivity testing of Mycoplasma bovis isolates derived from Western Canadian feedlot cattle. Microorganisms 8:124. doi:10.3390/microorganisms8010124 31963269 PMC7022776

[35] Hasoon MF, Jarocki VM, Mohammed MH, Djordjevic SP, Yip HYE, Carr M, Khabiri A, Azari AA, Amanollahi R, Jozani RJ, Carracher B, Mollinger J, Deutscher AT, Hemmatzadeh F, Trott DJ. 2023. Antimicrobial susceptibility and molecular characteristics of Mycoplasma bovis isolated from cases of bovine respiratory disease in Australian feedlot cattle. Vet Microbiol 283:109779. doi:10.1016/j.vetmic.2023.109779 37257307

[36] Whithear KG. 1983. Isolation of Mycoplasma alkalescens from cases of polyarthritis in embryo-transplant calves. Aust Vet J 60:191–192. doi:10.1111/j.1751-0813.1983.tb05964.x 6626069

[37] Kokotovic B, Friis NF, Ahrens P. 2007. Mycoplasma alkalescens demonstrated in bronchoalveolar lavage of cattle in Denmark. Acta Vet Scand 49:2. doi:10.1186/1751-0147-49-2 17204146 PMC1766361

[38] Dellinger JD, Jasper DE, Ilić M. 1977. Characterization studies on mycoplasmas isolated from bovine mastitis and the bovine respiratory tract. Cornell Vet 67:351–360. 326481

[39] Rosenfeld LE, Hill MW. 1980. The isolation of Mycoplasma alkalescens from an aborted bovine foetus. Aust Vet J 56:350. doi:10.1111/j.1751-0813.1980.tb05755.x 7437113

[40] Hirose K, Kobayashi H, Ito N, Kawasaki Y, Zako M, Kotani K, Ogawa H, Sato H. 2003. Isolation of Mycoplasmas from nasal swabs of calves affected with respiratory diseases and antimicrobial susceptibility of their isolates. J Vet Med B Infect Dis Vet Public Health 50:347–351. doi:10.1046/j.1439-0450.2003.00681.x 14535934

[41] Uemura R, Sueyoshi M, Nagatomo H. 2010. Antimicrobial susceptibilities of four species of Mycoplasma isolated in 2008 and 2009 from cattle in Japan. J Vet Med Sci 72:1661–1663. doi:10.1292/jvms.10-0165 20710124

[42] Hudson JR, Etheridge JR. 1963. A new type of pleuropneumonia‐like organism (Pplo) from the nose of cattle. Aust Veterinary J 39:1–5. doi:10.1111/j.1751-0813.1963.tb04166.x

[43] Manso-Silván L, Tardy F, Baranowski E, Barré A, Blanchard A, Breton M, Couture C, Citti C, Dordet-Frisoni E, Dupuy V, Gaurivaud P, Jacob D, Lemaitre C, Nikolski M, Nouvel L-X, Poumarat F, Thébault P, Theil S, Thiaucourt F, Sirand-Pugnet P. 2013. Draft genome sequences of Mycoplasma alkalescens, Mycoplasma arginini, and Mycoplasma bovigenitalium, three species with equivocal pathogenic status for cattle. Genome Announc 1:e00348-13. doi:10.1128/genomeA.00348-13 23766408 PMC3707579

[44] National Academies of Sciences, Engineering, and Medicine. 2016. Nutrient requirements of beef cattle. 8th ed. The National Academies Press, Washington, DC.

[45] Kozich JJ, Westcott SL, Baxter NT, Highlander SK, Schloss PD. 2013. Development of a dual-index sequencing strategy and curation pipeline for analyzing amplicon sequence data on the MiSeq Illumina sequencing platform. Appl Environ Microbiol 79:5112–5120. doi:10.1128/AEM.01043-13 23793624 PMC3753973

[46] Langmead B, Salzberg SL. 2012. Fast gapped-read alignment with Bowtie 2. Nat Methods 9:357–359. doi:10.1038/nmeth.1923 22388286 PMC3322381

[47] Martin M. 2011. Cutadapt removes adapter sequences from high-throughput sequencing reads. EMBnet j 17:10. doi:10.14806/ej.17.1.200

[48] Bolyen E, Rideout JR, Dillon MR, Bokulich NA, Abnet CC, Al-Ghalith GA, Alexander H, Alm EJ, Arumugam M, Asnicar F, et al.. 2019. Reproducible, interactive, scalable and extensible microbiome data science using QIIME 2. Nat Biotechnol 37:852–857. doi:10.1038/s41587-019-0209-9 31341288 PMC7015180

[49] Robeson MS 2nd, O’Rourke DR, Kaehler BD, Ziemski M, Dillon MR, Foster JT, Bokulich NA. 2021. RESCRIPt: reproducible sequence taxonomy reference database management. PLoS Comput Biol 17:e1009581. doi:10.1371/journal.pcbi.1009581 34748542 PMC8601625

[50] Callahan BJ, McMurdie PJ, Rosen MJ, Han AW, Johnson AJA, Holmes SP. 2016. DADA2: high-resolution sample inference from Illumina amplicon data. Nat Methods 13:581–583. doi:10.1038/nmeth.3869 27214047 PMC4927377

[51] McDonald D, Jiang Y, Balaban M, Cantrell K, Zhu Q, Gonzalez A, Morton JT, Nicolaou G, Parks DH, Karst SM, Albertsen M, Hugenholtz P, DeSantis T, Song SJ, Bartko A, Havulinna AS, Jousilahti P, Cheng S, Inouye M, Niiranen T, Jain M, Salomaa V, Lahti L, Mirarab S, Knight R. 2024. Greengenes2 unifies microbial data in a single reference tree. Nat Biotechnol 42:715–718. doi:10.1038/s41587-023-01845-1 37500913 PMC10818020

[52] Bisanz JE. 2018. qiime2R: importing QIIME2 artifacts and associated data into R sessions. Version 099 13

[53] Chen S, Zhou Y, Chen Y, Gu J. 2018. fastp: an ultra-fast all-in-one FASTQ preprocessor. Bioinformatics 34:i884–i890. doi:10.1093/bioinformatics/bty560 30423086 PMC6129281

[54] Wood DE, Lu J, Langmead B. 2019. Improved metagenomic analysis with Kraken 2. Genome Biol 20:257. doi:10.1186/s13059-019-1891-0 31779668 PMC6883579

[55] Jolley KA, Bray JE, Maiden MCJ. 2018. Open-access bacterial population genomics: BIGSdb software, the PubMLST.org website and their applications. Wellcome Open Res 3:124. doi:10.12688/wellcomeopenres.14826.1 30345391 PMC6192448

[56] Bushnell B. 2014. BBtools software package. Available from: https://sourceforge.net/projects/bbmap

[57] Alcock BP, Huynh W, Chalil R, Smith KW, Raphenya AR, Wlodarski MA, Edalatmand A, Petkau A, Syed SA, Tsang KK, et al.. 2023. CARD 2023: expanded curation, support for machine learning, and resistome prediction at the comprehensive antibiotic resistance database. Nucleic Acids Res 51:D690–D699. doi:10.1093/nar/gkac920 36263822 PMC9825576

[58] Wick R, Menzel P. 2021. Filtlong. Vv0.2.1. GitHub. Available from: https://github.com/rrwick/Filtlong

[59] Li H. 2016. Minimap and miniasm: fast mapping and de novo assembly for noisy long sequences. Bioinformatics 32:2103–2110. doi:10.1093/bioinformatics/btw152 27153593 PMC4937194

[60] Kolmogorov M, Yuan J, Lin Y, Pevzner PA. 2019. Assembly of long, error-prone reads using repeat graphs. Nat Biotechnol 37:540–546. doi:10.1038/s41587-019-0072-8 30936562

[61] Anonymous. 2018. Medaka. Oxford Nanopore Technologies. Available from: https://github.com/nanoporetech/medaka

[62] Jain C, Rodriguez-R LM, Phillippy AM, Konstantinidis KT, Aluru S. 2018. High throughput ANI analysis of 90K prokaryotic genomes reveals clear species boundaries. Nat Commun 9:5114. doi:10.1038/s41467-018-07641-9 30504855 PMC6269478

[63] Grant JR, Enns E, Marinier E, Mandal A, Herman EK, Chen C, Graham M, Van Domselaar G, Stothard P. 2023. Proksee: in-depth characterization and visualization of bacterial genomes. Nucleic Acids Res 51:W484–W492. doi:10.1093/nar/gkad326 37140037 PMC10320063

